# Insights into the dynamics between viruses and their hosts in a hot spring microbial mat

**DOI:** 10.1038/s41396-020-0705-4

**Published:** 2020-07-13

**Authors:** Jessica K. Jarett, Mária Džunková, Frederik Schulz, Simon Roux, David Paez-Espino, Emiley Eloe-Fadrosh, Sean P. Jungbluth, Natalia Ivanova, John R. Spear, Stephanie A. Carr, Christopher B. Trivedi, Frank A. Corsetti, Hope A. Johnson, Eric Becraft, Nikos Kyrpides, Ramunas Stepanauskas, Tanja Woyke

**Affiliations:** 1 Department of Energy Joint Genome Institute, Berkeley, CA USA; 2 Environmental Genomics and Systems Biology, Lawrence Berkeley National Laboratory, Berkeley, CA USA; 3 Civil and Environmental Engineering, Colorado School of Mines, Golden, CO USA; 4 Hartwick College, Oneonta, NY USA; 5 German Research Centre for Geosciences GFZ, Potsdam, Germany; 6 University of Southern California, Los Angeles, CA USA; 7 California State University Fullerton, Fullerton, CA USA; 8 University of North Alabama, Florence, AL USA; 9 Bigelow Laboratory for Ocean Sciences, East Boothbay, ME USA; 10 University of California, Merced, CA USA; 11 Present Address: AnimalBiome, Oakland, CA USA

**Keywords:** Bacteriophages, Metagenomics

## Abstract

Our current knowledge of host–virus interactions in biofilms is limited to computational predictions based on laboratory experiments with a small number of cultured bacteria. However, natural biofilms are diverse and chiefly composed of uncultured bacteria and archaea with no viral infection patterns and lifestyle predictions described to date. Herein, we predict the first DNA sequence-based host–virus interactions in a natural biofilm. Using single-cell genomics and metagenomics applied to a hot spring mat of the Cone Pool in Mono County, California, we provide insights into virus–host range, lifestyle and distribution across different mat layers. Thirty-four out of 130 single cells contained at least one viral contig (26%), which, together with the metagenome-assembled genomes, resulted in detection of 59 viruses linked to 34 host species. Analysis of single-cell amplification kinetics revealed a lack of active viral replication on the single-cell level. These findings were further supported by mapping metagenomic reads from different mat layers to the obtained host–virus pairs, which indicated a low copy number of viral genomes compared to their hosts. Lastly, the metagenomic data revealed high layer specificity of viruses, suggesting limited diffusion to other mat layers. Taken together, these observations indicate that in low mobility environments with high microbial abundance, lysogeny is the predominant viral lifestyle, in line with the previously proposed “Piggyback-the-Winner” theory.

## Introduction

Viruses shape phylogenetic and functional diversity of bacterial and archaeal communities [1, 2]. Our knowledge of viruses is rapidly increasing as a result of advances in computational methods for virus DNA/RNA sequence detection, which has enabled the development of large databases of complete viral genomes and viral proteins [3–6]. To fully understand the effects of viruses on local and global ecosystems, such as the control of host growth dynamics [7] or host-cell reprogramming through auxiliary metabolic genes [8, 9], it is critical to establish host–virus linkages for viruses found in a given environment.

Most viral sequences are currently not associated with any host, and for viruses with a known host, their full host range is usually unknown, hampering ecological and evolutionary insights [10]. Likewise, there are various bacterial and archaeal candidate phyla that have not yet been linked to any known viruses [6, 11]. Multiple computational approaches have been used to predict virus–host linkages in genome sequence data. For example, algorithms for detection of prophages in bacterial genomes [11–15] or protein sequence-based machine learning [16] enabled the detection of the first viruses for several uncultured bacterial phyla. However, these approaches are limited to viral sequences that are integrated into the host genome. Clustered Regularly Interspaced Short Palindromic Repeats (CRISPRs) in microbial genomes store short sequences from previous viral infections and they can be inferred bioinformatically at the species level [17], but only a limited number of bacterial lineages use this virus-defense system [18]. In addition, tRNA sequences acquired by viruses during host infection [5], along with host and virus similarities of oligonucleotide signatures [19, 20], can be used to link viruses with uncultured bacterial or archaeal hosts, but with only limited accuracy. Recently, it has been suggested that similar DNA methylation patterns may also allow the assignment of viruses to hosts within metagenomes, although this approach remains to be validated [21]. While computational methods are powerful, the necessary sequence features are not always present in both virus and host genomes [5, 18], and predicted associations should still be validated experimentally when possible [22].

Studies of viruses in isolated microbes used to be the gold standard for providing experimental evidence of infective strategies [23], but many hosts cannot be cultivated [24], and even intensively studied bacteria, such as those found in the human gut, often lack any isolated viruses [10]. Single-cell genomics represents a unique opportunity to link viruses and hosts with experimental evidence, because a certain portion of cells collected directly from an environment contain viruses in the cell or attached to the cell [25, 26]. This approach has shed light on important aspects of viral biology, such as horizontal gene transfer [27], the ability of viruses to reprogram their host’s energy metabolism [28] and micro-diversity within viral genomes [29]. Single-cell genomics in the context of studying viruses has been successfully applied to a variety of habitats, ranging from seawater [30] to hot springs [31].

It has been estimated that 40–80% of microbial cells on Earth reside in biofilms [32], but surprisingly they are understudied by single-cell genomics. Biofilms are formed by aggregates of microorganisms in which cells are embedded in a self-produced matrix of extracellular polymeric substances that are adherent to each other and/or a surface [33]. Biofilms, which develop in a liquid–solid interface and contain layered organization of microorganisms, are called microbial mats [34]. Microbial mats are found across the planet in a variety of habitats [35–40]. Their laminated structure contains bacteria, archaea, and eukaryotes that work together, often symbiotically, sharing and cycling nutrients and energy [38, 41]. Often driven by photosynthesis at their surface and chemosynthesis at their base, microbial mat structures are a key component of the living world that provide us with a snapshot of how microorganisms work together in a complex, ordered community to propagate and ensure their survival. Microbial mats can also serve as potent biosignatures of life on Earth. As an example, stromatolites, laminated accretionary structures found throughout the rock record can be biogenic and are likely the mineralized, fossil record of microbial mats across time thus informing the evolution of life [39, 42, 43].

There are only a few single-cell genomic studies about bacteria in natural biofilms [44–46], in which viruses were not considered, leaving a knowledge gap in host–virus interactions in the microbiomes of these ecosystems. Viruses in biofilms have been typically studied separately from their hosts as purified viral particles, leaving CRISPR linkage as the only way of connecting them with their host [47]. The most common method for analyzing host–virus interactions in biofilms is computational modeling based on data from laboratory experiments performed with cultured bacteria-phage pairs [48–51], which complicates its application to complex natural biofilms. On the other hand, computational predictions have revealed important information about host–virus interactions in biofilms. Such work points out that host–virus interactions depend on a variety of factors, such as biofilm species composition, structural heterogeneity [52], and metabolic activity of bacteria in different layers of the biofilms [53]. It has also been suggested that viruses may enhance biofilm formation through induction of polysaccharide production [54, 55]. While computational predictions are the first step for interpreting dynamics of viral infection in biofilm microbial communities, experimental studies on naturally occurring biofilms are instrumental in order to both further our understanding of viral infections in complex natural biofilms and refine computational projections [56].

We characterized the host–virus linkages in a laminated microbial mat within the geothermal pool “Cone Pool” (Long Valley Caldera, CA, USA). We combined single-cell genomics with bulk shotgun metagenomics to predict different aspects of host–virus interactions, including viral host range, viral genome copy number compared to its host, and diffusion of viruses across the mat layers. Taken together, our data point to a narrow host range and a low level of active viral replication in this microbial mat, indicative of a higher prevalence of temperate viruses in a lysogenic infection stage, and limited diffusion of viruses within the mat system. The Cone Pool mat has many functional similarities to other microbial mats, such as limited diffusion of viruses into deeper layers and diurnal vertical migration of redox gradients [57], which suggests that the results of this study might be more broadly applicable to other structured biofilms in different environments.

## Methods

### Sample collection and processing

**Fig. 1:**
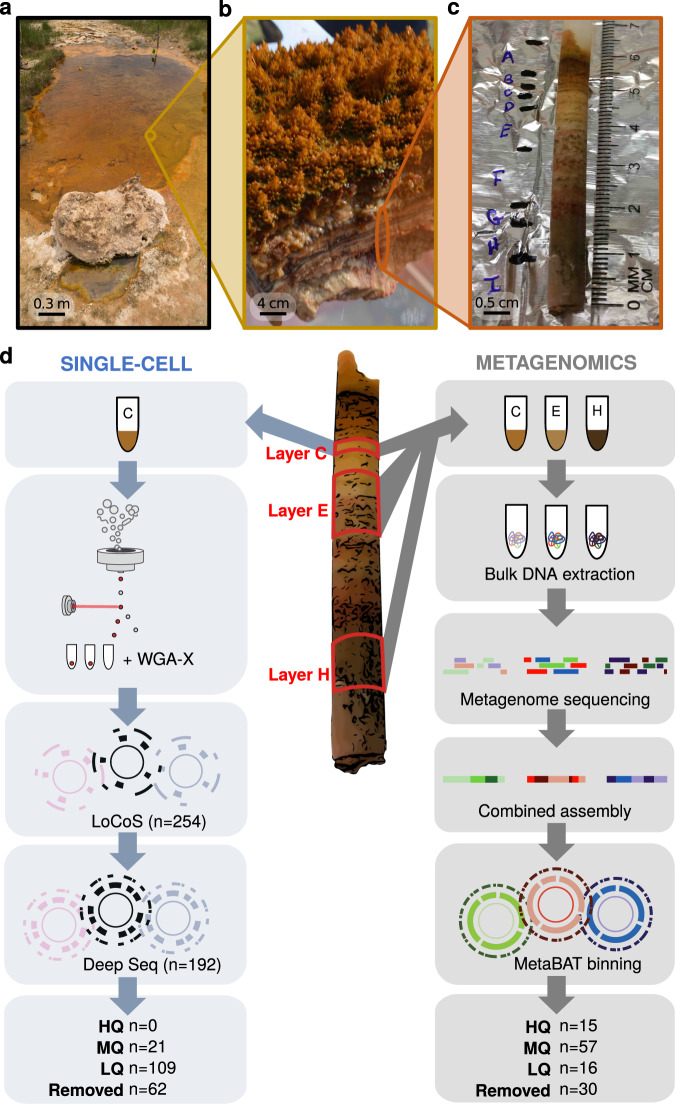
Experimental workflow. **a** Overview of Cone Pool hot spring; **b** section through microbial mat, showing dendritic cones and layers of the mat; **c** delineation of layers of the mat; and **d** Sequencing workflow for single-cell genomics (left, layer C) and shotgun metagenomics (right, layers C, E, and H). The numbers at the bottom of the figure indicate the number of resulting high quality (HQ), medium quality (MQ) and low quality (LQ) genomes, as based on MISAG/MIMAG standards.

Samples from a laminated microbial mat at a geothermal pool (“Cone Pool”), located in the Little Hot Creek geothermal spring area within the Long Valley Caldera, California (37.6905833° N, 118.844417° W), were collected on the 15^th^ of August 2015, under the umbrella of the Microbial Dark Matter (MDM) Phase II study, an extension of the Genomic Encyclopaedia of Bacteria and Archaea MDM project (GEBA-MDM; [58]). An intact, submerged, dendrolitic cone, and the laminated mat beneath, was cored using a sterile drinking straw from the edge of the pool. The straw was shipped to the Colorado School of Mines, Golden, Colorado, on ice, and stored at 4 °C. Layers of the mat were delimited based on color and consistency, designated “A” through “I” by slicing through the straw using a sterile scalpel (Fig. 1). The layers “B” to “H” were extruded from the straw casing and divided for DNA extraction.

DNA for metagenomic and 16S rRNA gene amplicon sequencing was extracted from 0.25 g of each layer using the Xpedition™ Soil/Fecal DNA MiniPrep kit (Zymo Research Corp.), which uses bead beating based lysis previously shown to break capsids of dsDNA viruses [59]. The remainder of layer C was divided into replicates (~0.1–0.5 g each), submerged in 1 ml PBS, vortexed for 30 s, and centrifuged for 30 s at 2000 rpm. The 1 ml supernatant was then mixed with 100 µL of 0.2 μm filter sterilized GlyTE (20 ml 100× TE Buffer pH 8.0, 60 ml deionized water, 100 ml molecular grade glycerol), incubated at room temperature for 1 min, and then stored at −80 °C for downstream single-cell analysis.

### Bacterial/archaeal composition via amplicon sequencing

The 16S rDNA amplicons of the regions V4 and V5 were obtained using primers 515F-Y (5′-GTGYCAGCMGCCGCGGTAA) and 926R (5′-CCGYCAATTYMTTTRAGTTT) [60] sequenced on an Illumina MiSeq sequencer in 2 × 300 bp run mode. Amplicons from the layer D were not sequenced due to poor DNA amplification. The sequence analysis was performed on 3.6 × 10^5^ (±1.1 × 10^5^) sequences per sample using the JGI iTagger v2.0 pipeline [61] that cluster sequences into operational taxonomic units (OTU) with 97% sequence similarity in the USEARCH software suite [62] and performs ecological analyses in QIIME [63] using RDP classifier v2.5 [64]. The purpose of 16S rDNA amplicon analysis was to select mat layers containing the highest abundance of understudied bacterial phyla [58] for subsequent shotgun metagenomic and single-cell genomic analysis.

### Bulk shotgun metagenome

Genomic DNA from layers C, E, and H was sequenced on the Illumina HiSeq-2500 platform (libraries with 300 bp inserts) at the Department of Energy Joint Genome Institute in 2 × 150 bp mode resulting in 101 × 10^6^, 63 × 10^6^ and 83 × 10^6^ reads from layers C, E, and H, respectively (JGI sequencing project Gold IDs: Gp0147099, Gp0147100 and Gp0147101, respectively). Reads were trimmed and screened for common laboratory contaminants with BBTools v.37 (Bushnell [65], http://bbtools.jgi.doe.gov) and the sequencing errors were corrected by bfc v.181 [66] with the following parameters: “-s 10 g -k 21”. Mate-pair reads were assembled using SPAdes v.3.10.0 [67] with specified kmers 21, 33, 55, 77 and -meta flag. The co-assembly of all three metagenomic datasets was annotated using the IMG system [68, 69] and is publicly available under IMG taxon ID 3300022548. Using the contigs of the combined assembly, metagenome-assembled genomes (MAGs) were created by combining initial sets of genome bins from seven different binning approaches: (1) MaxBin v1.4.5 [70] using the universal 40 marker gene set and (2) the 107 marker gene set; (3) MaxBin v2.2.4 [71] with default parameters; (4) MetaBAT1 v0.32.5 [72] using the “super-specific” parameter and (5) “super-sensitive” parameter; (6) MetaBAT2 v2.12.1 [73] using default parameters; and (7) CONCOCT v0.4.0 [74] using default parameters. All binning methods used a minimum contig size of 3000 bp. Bins generated using the seven methods were used as input to DAS Tool v1.1.0 [75], which was run with default parameters to generate the final MAG set.

### Single amplified genomes

Single amplified genomes (SAGs) were generated from layer C at the Single Cell Genomics Center at Bigelow Laboratory for Ocean Sciences. Briefly, single cells were isolated with fluorescence-activated cell sorting (FACS), lysed with a combination of freeze-thawing and alkaline lysis, and genomic DNA was amplified with WGA-X [76]. Barcoded libraries were created using Nextera XT (Illumina) following standard protocols. Low-coverage shotgun sequencing (LoCoS) and de novo assembly was carried out at Bigelow Laboratory for Ocean Sciences as previously described [76]. SAGs which had at least 50,000 trimmed reads, 1000 normalized reads, and a total assembly size of at least 50 kb from LoCoS were selected for deeper sequencing (*n* = 192). Deep sequencing of libraries was performed at the DOE Joint Genome Institute on the Illumina NextSeq platform in 2 × 150 bp mode. Raw reads were filtered for quality and contamination with BBTools v.37 (Bushnell [65], http://bbtools.jgi.doe.gov), then BBTools components BBNorm and Tadpole were used for read normalization and error correction prior to assembly with SPAdes (v3.9.0; --phred-offset 33 --sc -k 22,55,95 --12) [77]. After assembly, 200 bp was trimmed from contig ends, contigs <2 kbp in length or with read coverage <2 were discarded, and annotation was carried out according to IMG standard protocols [68, 69].

### Taxonomic classification of the host genomes

The overall quality of SAGs and MAGs was categorized according to published standards [78]. CheckM v1.0.8 using lineage-specific workflow [79] was used to estimate completeness and contamination. Genomes with more than 10% estimated contamination (*n* = 62) were excluded from further analysis.

A set of 56 universal single-copy marker proteins [80, 81] was used to build a phylogenetic tree for the newly generated SAGs and MAGs and a representative set of bacteria and archaea based on all publicly available microbial genomes in IMG/M ([68]; genomes accessed in April 2018) and about 8000 MAGs from the Genome Taxonomy Database (GTDB, [82], downloaded 18 October 2017). Marker proteins were identified with hmmsearch (version 3.1b2, hmmer.org) using a specific HMM for each of the markers. For every marker protein, alignments were built with MAFFT (v7.294b, [83]) and subsequently trimmed with BMGE using BLOSUM30 [84]. Single protein alignments were then concatenated resulting in an alignment of 10,866 sites. Maximum likelihood phylogenies were inferred with FastTree2 using the options: -spr 4 -mlacc 2 -slownni -lg [85]. In the following step, a subtree was built that employed the above described methods but included only query genomes and reference genomes from query-genome containing clades in the initial tree. The genomes were classified at the phylum level using the naming system of the National Center for Biotechnology Information taxonomy [86].

In addition, all genomes were classified with the GTDB Toolkit v0.1.0 [87] according to the GTDB taxonomy, which was created to standardize microbial taxonomy according to genomic information [88].

Finally, all MAGs and SAGs were clustered by Mash v1.1 [89] using a 95% average nucleotide identity (ANI) cutoff to approximate a species-level resolution.

### Putative viral sequence detection

VirSorter [3] and comparison to the IMG/VR database [6] were used to detect viral sequences in SAGs, MAGs, and the unbinned fraction of metagenomic contigs.

VirSorter was used on all contigs at least 2 kb in length, retaining predictions from categories 1 and 2 (fully viral contigs), and 4 and 5 (integrated viruses). Contigs in which bitscores of pfam hits were higher than bitscores of viral hits were not considered as viral contigs.

In the next step, all sequences were queried against the full IMG/VR database (version IMG_VR_2018-01-01_3) with BLAST [90]. Hits where alignment length was at least 70% of the query or subject sequence length (whichever was shorter) were retained (at the “detection” threshold, [91]), and all overlapping and adjacent hit regions of each query contig were merged into consensus coordinate range(s) with the R package plyranges [92]. Single best hits with at least 90% identity and 75% alignment coverage to IMG/VR were assigned with the IMG/VR Viral Cluster ID (“assignment” threshold, [91]); otherwise the contigs were annotated as a novel virus.

Results from the two prediction approaches were combined to extract viral sequences. The coordinate range(s) predicted as viral by each method were merged with the R package plyranges [92], to obtain the most inclusive estimates of viral sequence. The final set of viral sequences was clustered with MUMmer v3.23 [93], requiring at least 95% ANI over at least 85% of the length of the shorter of the two sequences to add sequences to a cluster, in accordance to community standards [16].

An additional curation step was performed for viral contigs within MAGs in order to remove viral contigs erroneously binned together with a bacterial genome. For that reason, only integrated viruses containing flanking bacterial sequences (VirSorter categories 4 and 5) and viral contigs clustering with other integrated viruses were retained; otherwise they were assigned to the unbinned fraction which represented 40% of the reads.

### CRISPR-based host–virus linking

The host–virus pairings detected in SAGs and MAGs were tested for their consistency with clustered regularly interspaced short palindromic repeat (CRISPR)-based linking prediction [94]. CRISPRs in host genomes were identified using CRT v1.1 [17] with script modifications as used in the IMG/M [68], CRISPRCasFinder [95] and CRISPRDetect [96] and 100% identical spacers detected by these three programs in the genomes belonging to the same hosts species (95% ANI) were de-replicated by cd-hit v4.8.1 [97] and matched against all viral genomes found in the Cone Pool using blastn [90], where only identical hits over the complete length of a CRISPR spacer were scored as a positive match.

### Host–virus ratios

The host–virus genome coverage ratio was assessed by mapping the metagenomic reads from the layers C, E and H to a reference database containing host and virus genomes obtained in the previous steps by BBMap (Bushnell [65], https://sourceforge.net/projects/bbmap/) using the default settings. From the viruses that formed sequence-similarity clusters (see above), only the longest viral contig was selected as a cluster representative for mapping. From the host genomes grouped by ANI (see above), the genome with the highest genome completeness and the lowest contamination (estimated by CheckM) was selected as the cluster representative for mapping. The number of reads per sample used for mapping was normalized to the lowest number of reads per sample obtained for the three layers. Only the genomes with reads distributed across more than 75% of their genome length (“assignment” threshold, [91]) were considered as positive hits.

## Results and discussion

### Cone Pool microbial community data through amplicons, SAGs and metagenomes

To first assess the overall microbial community structure of the Cone Pool hot spring microbial mat, 16S rRNA gene amplicon analysis was performed on the mat layers B, C, E, F, G and H (Fig. 1), exclusive of the cone tip (layer A), which has previously been published [57]. The amplicon data yielded 440 OTUs, of which 24 had average abundances higher than 1% (Supplementary Fig. S1). The six mat layers differed remarkably in their microbial composition. Layer B (Fig. 1) was dominated by aquatic thermophiles, as was the cone tip previously analyzed from the same sampling site [57]. In contrast, the lower layers had a higher proportion of candidate phyla, including *Aminicenantes* (OP8), *Microgenomates* (OP11), and *Edwardsbacteria* (AC1). Layer C was selected for single-cell genomics analysis to capture some of the candidate phyla representatives while minimizing any potential challenges that might occur during cell sorting due to accumulation of calcium carbonate in the lower layers of the mat. Layers C, E, and H, providing three reference points across the mat, were selected for shotgun metagenomic sequencing.

**Fig. 2:**
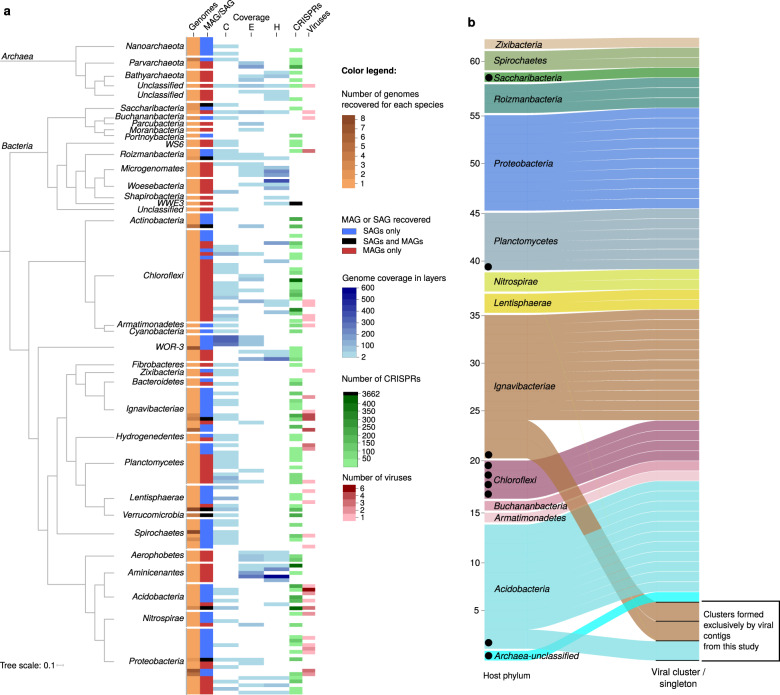
Summary of microbes and viruses found in this study. **a** Bacterial and archaeal species recovered by metagenomics and single-cell analysis. Phylum-level cluster representatives are displayed in a phylogenetic tree based on concatenated alignment of 56 universal single copy marker proteins. Each row represents a species based on 95% ANI. The first two columns represent the number of genomes in each species cluster and the source of the genomes of the given species (SAGs only, MAGs only or both MAGs and SAGs). The following three columns show read coverage of each species in metagenomics samples from layers C, E and H. The last two columns indicate the number of CRISPR spacers and of viral contigs detected for each species. **b** Alluvial plot of virus and host connection. The left panel represents host species, colored by phylum, and the right panel shows viral clusters separated by horizontal black lines and singletons. The black dots indicate viruses detected on MAGs, while other viruses were detected on SAGs. Full results of this analysis, including the viruses with unknown host information, are shown in Supplementary Fig. S3.

Single-cell genomics from layer C generated 254 SAGs; 192 SAGs met LoCoS selection criteria for deep sequencing, from which 130 SAGs passed our minimum genome quality thresholds of genome completeness, contamination and taxonomic classification as described in the “Methods” (Fig. 1, Supplementary Table S1). The 130 SAG assemblies averaged 1 ± 0.5 Mbp in size with a 34.1 ± 16.0% estimated genome completeness (range 2.7–74.9%), totaling 21 medium quality and 109 low quality genomes, as based on MISAG standards [78]. Binning contigs from the co-assembly of metagenomes from layers C, E, and H resulted in 88 MAGs which averaged 2 ± 1.2 Mbp in size with 73 ± 19% estimated genome completeness (range 25.4–99.0%), consisting of 15 high-quality, 57 medium-quality, and 16 low-quality genomes, based on MIMAG standards ([78]; Fig. 1, Supplementary Table S1). ANI-based clustering (>95% ANI, [98]) of the 130 SAGs and 88 MAGs resulted in 144 bacterial and 15 archaeal ANI-based, nominal species-level groups, distributed across 36 phyla, mostly *Proteobacteria*, *Chloroflexi*, *Ignavibacteriae* and *Planctomycetes* (genome classification based on 56 markers genes, see “Methods”, Fig. 2a, Supplementary Table S1).

### Host–virus links in the Cone Pool mat microbiome

To make inferences about different aspects of host–virus interactions in the Cone Pool mat, virus detection tools were applied to SAGs and metagenome contigs. Using two different approaches for viral sequence detection (VirSorter and comparison to IMG/VR), 385 putative viral contigs were identified: 53 viral contigs (14%) were detected in 34 SAGs, 9 viral contigs were found (2%) in 9 MAGs, and 323 (84%) in the unbinned assembled metagenomes (Supplementary Table S2, Supplementary Fig. S2). Eighty-eight out of 385 detected viruses (23%) matched viruses from IMG/VR forming 52 groups with up to 5 viruses (Supplementary Fig. S3). The low number of matches to IMG/VR (at the time of analysis containing ~730,000 viruses) highlights the uniqueness of this sampling site, with a paucity of existing relatives in the database. The matched IMG/VR contigs mainly came from different thermal spring environments with some similarities to Cone Pool (Supplementary Table S2), but none of them were associated with a host in IMG/VR. Remarkably, the single-cell genomes from this study uncovered hosts for four IMG/VR contigs that had no previous host information.

Twenty-six percent of SAGs in this study contained 1–6 viral contigs (originating from attached/intracellular virions or integrated viruses), which was similar to infection rates reported for SAGs from surface ocean bacterioplankton [30]. When considering the results across host taxa, viruses were detected in 14 out of 36 host phyla, and in 21% of host species (95% ANI) detected in this study (Fig. 2a). *Acidobacteria* was the phylum in which viruses were detected most frequently (7 out of 9 genomes had a virus). In contrast, no viruses were associated with *Aminicenantes, Nanoarchaeota*, and WOR-3, despite these taxa being represented by multiple SAGs or MAGs. To summarize, we detected a total of 62 viruses in 43 different SAGs or MAGs, which sequence similarity-based clustering reduced to 59 viruses and 34 host species (Fig. 2b). These included 56 singletons and three clusters of two sequences each, for which both members were always linked to the same host species (Fig. 2b). The small size of these viral clusters is not sufficient to assess the virus–host range in Cone Pool mat, but the wide diversity of detected viruses suggests that there is no dominant virus targeting multiple host species. One might hypothesize that a laminated microbial mat has the potential to select for broader host ranges of resident viruses, since viruses and bacteria exist in proximity over the long-term with limited diffusion between the layers [52]. However, our single-cell genomic data from Cone Pool does not support this scenario.

### CRISPR-based linking

The detection of viral contigs in SAGs is evidence of ongoing viral infection in the collected single cells. Additional host–virus links can be obtained by analyzing the presence of CRISPRs in the host genomes that can indicate past viral infections. CRISPRs were detected in 43% of host genomes in this study (Fig. 2a). From the 385 viruses detected in the Cone Pool, only three viruses were linked to their hosts by CRISPR. These hosts belonged to *Aminicenantes, Lentisphaerae,* and *Portnoybacteria* phyla and their CRISPR-associated viruses were found in the unbinned fraction. The 62 viruses which were detected on 43 SAGs or MAGs were not targeted by CRISPRs found in these genomes, which suggests that these hosts have not yet built defenses against these viruses. The low CRISPR-based links in the Cone Pool highlights the utility of using single-cell genomics for linking viruses with their hosts.

### Lack of active replication of viruses associated with single cells

To gain insights into the putative interactions of viruses and hosts within the microbial mat environment, we were interested in the level of active viral replication. Viral contigs without flanking bacterial sequences in a SAG do not necessarily represent attached or intracellular virions during active infection. Due to the fragmentation of genome assembly from short reads, inactive integrated viruses might be found without the flanking bacterial sequences [10]. In the present study, only four SAGs contained integrated prophages with flanking bacterial sequences (Supplementary Table S2). To assess active viral replication in the Cone Pool microbial mat, we applied two different methodologies.

First, we applied an approach proposed by Labonté et al. [30], which relies on the correlation between the amount of DNA template and the speed of whole-genome amplification (WGA). If a SAG contains an actively replicating virus, WGA proceeds relatively fast (expressed as low crossing point (Cp) values of real-time WGA kinetics), but results in low host genome completeness, which is a consequence of: (a) large fraction of DNA available to WGA being viral, and (b) partial degradation of host genome by the lytic infection. The identification of SAGs with actively replicating viruses is based on comparison with SAGs with no viruses from the same experiment. In the Cone Pool data, none of the SAGs containing viral contigs had significantly lower Cp values of the WGA-X reaction, nor significantly lower genome coverage compared to the SAGs without viral signal (Supplementary Fig. S4), indicating that the viruses in our single-cell dataset were not actively replicating in the host cell.

Second, according to Schulz et al. [99], genomes present in multiple copies in a collected MDA-enriched sample, in theory, could be identified (despite the WGA bias) by having read coverages that were hundreds of times higher than other contigs in the sample. This trend was not observed in our SAG dataset, which further suggests that the collected single cells did not contain actively replicating viruses (Supplementary Fig. S5).

### Low level of active viral replication across the mat layers

**Fig. 3:**
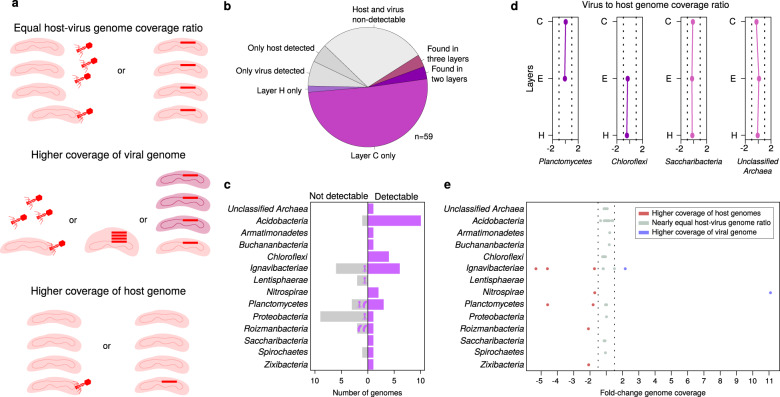
Host–virus genome read coverage ratios. **a** Possible scenarios for the interpretation of genome read coverage results. If there is a low rate of viral replication, we expect the genome coverage ratio of the virus and host to be nearly the same in a metagenome. Higher viral coverage could result from a higher number of virions compared to host cells, or more copies of the viral genome in each infected cell, but could also mean that a lysogenic virus has more than the single predicted host species. Higher coverage of a bacterial genome suggests that single-cell genomics captured a very rare infection event or that the virus infected only a subset of cells (i.e. only certain strains). **b** Detection of the 59 de-replicated host–virus pairings in the three layers. The gray portions indicate the pairs in which virus, host, or both genomes were below the detection threshold. For 35 pairings (purple), detection was possible in at least one of the layers (>75% of the genome length covered). **c** Number of host genomes in each phylum for which the host–virus genome coverage ratio could (Detectable) or could not (Not detectable) be calculated. Virus and host icons indicate which one from the host–virus pair was above the detection threshold. **d** Fold-change of host and virus genome coverage for the four pairs detected in two or three layers. The dashed line indicates the 1.5× fold-change range. Dots positioned on the right from the host-baseline in the middle indicate higher coverage of the viral genome, while on the left indicate lower coverage of the viral genome compared to the host. **e** Fold-change of host and virus genome coverage of all 35 host-virus pairs (dots) grouped by host phyla. The distribution of points relative to the *x*-axis is described in (**d**).

We also analyzed whether viruses were actively replicating in all samples from the Cone Pool microbial mat layers. Replication of viruses can be assessed by mapping metagenomic reads to host–virus pairs detected in SAGs obtained from the same sample, followed by the comparison of host and virus genome coverage [10]. Typically, metagenomic samples contain DNA from bacterial cells as well as from highly abundant intracellular viral particles (if their capsids are opened during DNA extraction). Moreover, active replication of viruses inside the bacterial cells can also be detected in the metagenome data [100]. Because DNA of hosts and viruses in metagenomic reads is not amplified by WGA, it provides a more robust estimate of genomic DNA copies of both viruses and their hosts on the community level. By applying such read recruitment, we assumed that if a virus has the same genome coverage as its host, it is not actively replicating (Fig. 3a). In contrast, actively replicating virus would have a higher genome coverage than their host, due to the additional viral genome copies either free in the cytoplasm of a virocell [101–103] or in newly formed viral particles released from the host cell (Fig. 3a). We mapped the metagenomic reads from Cone Pool microbial mat layers C, E and H to de-replicated pairs of 59 viruses and their 34 hosts. It was possible to calculate the host–virus genome coverage ratios for 35 of these inferred pairs; the remaining 24 pairs had coverages below the detection threshold (>75% of the genome length covered, Fig. 3b), from which three pairs were not detectable due to the low coverage of viruses and four pairs due to the low coverage of the host (Fig. 3c), but the genome coverages of their detectable counterparts were not exceptionally high compared to the average in the Cone Pool. The pairs below the detection threshold involved hosts from seven host phyla (Fig. 3c). Given that assessing host–virus relationships directly from metagenomic data in complex communities is typically limited to highly abundant bacteria or archaea and viruses with known predation patterns [7, 104], our ability to calculate host–virus coverage ratios for so many pairs highlights the utility of single-cell genomics for capturing rare host–virus pairings.

From the 35 detectable host–virus pairs, only four pairs were found across multiple layers. Genome coverage of hosts was similar to the genome coverage of their viruses (<1.5× fold difference), and this ratio was conserved across different layers (Fig. 3d). In total, 76% of all detectable pairs involved hosts and viruses with nearly equal genome coverages, indicating a low level of active viral replication, while also suggesting that induction of integrated viruses or active infection in the mat is not common, or was not occurring at the time of sampling (Fig. 3e). Alternatively, if some viruses were actively replicating, they did not form large numbers of progeny or only infected a small fraction of the available host cells, such that the host-to-virus genome coverage ratio looked similar at the population level in a given layer (Fig. 3a). This is in accordance with previous studies on microbial mats that were based on the counting of viral particles and bacterial cells by fluorescent microscopy [105, 106]. The study of Carreira et al. [106] performed on a photosynthetic microbial mat showed microscale (mm) and seasonal variation of the viruses-to-bacteria ratio, but viruses never outnumbered bacteria by orders of magnitude as is usually reported for the marine environment [107]. Our metagenomic read mapping analysis revealed eight viruses that had genome coverages lower than their hosts (up to 5.3× times lower, Fig. 3e). This suggests that these viruses were present only in a subset of cells of a given host species (Fig. 3a), e.g., in a susceptible strain or in a subpopulation of cells which has transiently lost immunity [108]. Lower genome coverage of a virus compared to its host could also be explained by host genome polyploidy [109]. However, polyploidy is common only in extremely large bacterial cells and these were not targeted by FACS in this study. Only two viruses had a genome coverage higher than that of their host (>1.5×). The coverage of one of these viruses was 11 times higher than its *Nitrospirae* host (Fig. 3e), indicating induction of integrated viruses, plasmid-like replication, lytic lifestyle, or existence of additional hosts, which remain uncovered (Fig. 3a).

Interestingly, each of the 35 inferred viruses was only detectable in those layers where its host was present, which indicates that these viruses likely resided in proximity to and/or within host cells. It is possible that integrated viruses were spontaneously induced in a small portion of cells to enhance biofilm integrity, and thus the resulting viral particles remained near the host cells from which they originated [110, 111]. However, this does not exclude the possibility of released virions penetrating adjacent layers, where they might perish without their hosts and thus remain undetectable [112].

### Mat viruses exhibit high layer specificity

**Fig. 4:**
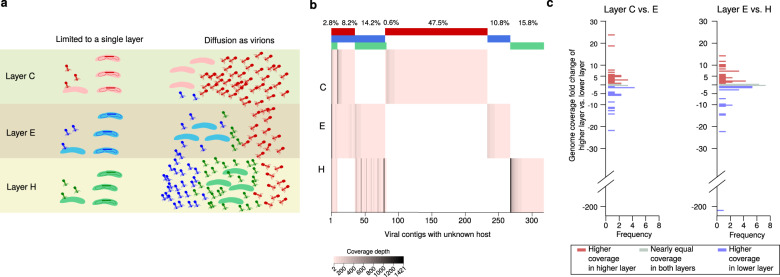
Predicted virus diffusion across the mat layers. **a** Possible scenarios of virus diffusion across the layers. Given that the layers differ by bacterial composition, a layer-specific viral composition suggests limited diffusion across the layers. If the viruses can move across layers, their abundances would vary across layers. **b** Genome coverage of the 323 virus sequences with no host information across the mat layers. The red, blue, and green stripes above the heatmap indicate whether the viruses were detected in one, two, or three layers, and the percentage above the stripes indicate the proportion of each of these groups. **c** Histograms of genome coverage fold-change of viruses detected in upper layer compared to the lower layer.

The analysis of the viral distribution in the mat layers described above was limited to the 35 viruses with identified hosts detectable in the metagenomic reads, pointing to only four viruses distributed across multiple mat layers (Fig. 3d). However, there were another 323 viral contigs detected in the unbinned fraction that could not be linked to any host (Supplementary Fig. S2). Given that the bacterial distribution in the Cone Pool mat is generally layer-specific (Supplementary Fig. S1, Fig. 2a), these viruses with unknown hosts could also be used for a more robust analysis of the viral composition and diffusion across the mat layers (Fig. 4a).

We mapped the metagenomic reads from the layers C, E, and H to the viral contigs from the unbinned fraction. We found that 75% of them were detected only in one layer (Fig. 4b), which confirms the high layer-specific viral composition of the Cone Pool microbial mat. Similar spatial distribution of viruses on a microscale was reported for the soil sampled in 1-cm resolution [113] and from the surface and inside of plant leaves [114].

The highest diversity of viruses was detected in layer C (Fig. 4b), which might be a consequence of this layer being close to the mat surface where it would be exposed to viruses from outside of the mat. This observation is in accordance with reports of higher viral counts on a microbial mat surface compared to the mat interior [53, 106]. Because there was no excessive accumulation of virions in the lower layers compared to upper layers in the Cone Pool microbial mat (Fig. 4c), gravity is not likely the principal force of virion diffusion in this mat; rather it is the tight virion/host-cell association in the spatial zonation of the mat that determines virion abundance. In addition, it has been shown that biofilm maturation and structural complexity are critical for protecting the bacteria against a continuous flux of phages from outside of the biofilm [115].

Interestingly, the metagenomic read mapping showed that the genome coverage of 93% of the viruses from the unbinned fraction was lower than the genome coverage of both MAGs and SAGs in this study. This likely means that the Cone Pool mat has only a small portion of actively replicating viruses. Taken together, it is likely that the low viral progeny number compared to host cells is an effective strategy for viral survival in the layers of the Cone Pool mat, as there is a limited supply of new hosts and a limited possibility to diffuse to other layers. This is in accordance with the “Piggyback-the-Winner” theory of viral infection stating that lysogeny is the predominant viral lifestyle in low mobility environments with high microbial abundance, where integrated viruses help their hosts to prevent infection by closely-related viruses which increases their ability to compete with other host species [116]. While integration of a virus into the host chromosome generally represents an extra energetic expense for the host, such expenses are probably insignificant in nutrient-rich and protective environments, such as a microbial mat [117]. Taken together, the predicted host–virus interactions in the Cone Pool mat are quite similar to the gut microbiome, which is generally characterized by a high concentration of bacterial cells and a high prevalence of lysogeny [118]. In comparison, aquatic environments with higher mobility of biomass are generally reported to have dynamic virus–host ratios [119], but this dynamics can decrease with sampling depth [120] or can have seasonal variations when switches between lytic and lysogenic cycles occur [121].

## Conclusions

By detecting viral contigs in both SAGs and MAGs from the Cone Pool microbial mat, we linked 59 viruses with 34 hosts, many of them belonging to taxonomic groups with no cultured representatives. Due to the complex bacterial/archaeal and viral composition of this mat and the limited direct detection of integrated viruses in host genome assemblies from this mat metagenomes, we infer that most of the host–virus pairings in this study could not have been obtained if not for the employed single-cell genomics methodology. This highlights the utility of the technique for linking viruses with their hosts to provide a deeper understanding of mat microbial ecology.

Single-cell genomics paired with metagenomic read recruitment provided insights into viral host range and distribution across the mat layers, as well as predicted viral lifestyle. While previous studies on bacterial biofilms have focused on computational predictions and were based on laboratory experiments with a limited number of cultivated phages and bacteria, this is the first study that reports detailed infection dynamics in a complex natural microbial mat for host–virus pairs with known identity. Our results point to a low rate of active viral replication in each layer and a limited spread of viral particles across the mat layers. This hints to different factors shaping the mat layers microbial composition, such as bacterial predation by nematodes [122] or seasonal variation in bacterial/archaeal metabolism [123]. While our work shed light on diversity of dsDNA and ssDNA viruses in microbial mats, further work might involve investigation of ssRNA viruses, as these have been found in abundance in other environments [124, 125] and could play a role in biofilm formation [54]. We believe that our observations can be expanded to other natural biofilms and contribute to the development of novel microbial dynamics prediction models for biofilms.

## Supplementary information

Supplementary Figure S1

Supplementary Figure S2

Supplementary Figure S3

Supplementary Figure S4

Supplementary Figure S5

Supplementary Table S1

Supplementary Table S2

## Data Availability

The sequences and the genome assemblies are accessible on Integrated Microbial Genomes and Microbiomes website https://img.jgi.doe.gov/ with IDs listed in Supplementary Table S1.
