# Supplementary material for: Insights into the dynamics between viruses and their hosts in a hot spring microbial mat
Source: ISME J. 2020 Jul 13;14(10):2527–41. doi: 10.1038/s41396-020-0705-4 (PMC7490370; doi:10.1038/s41396-020-0705-4)

**Supplementary Figure S1: Bacterial composition of the layers B, C, E, F, G and H.** The operational taxonomic units (OTUs) in the barplots in the panel **a** are ordered by their average proportion across the six layers (only OTUs with an average proportion >1% across the 6 samples are shown). In the separate bartplots in the panel **b**, the OTUs conserve the same colouring as in the panel **a** and the OTUs with no colour (white) indicate layer-specific OTUs not shows in the panel **a**. The OTUs with proportion < 1% are black-coloured.

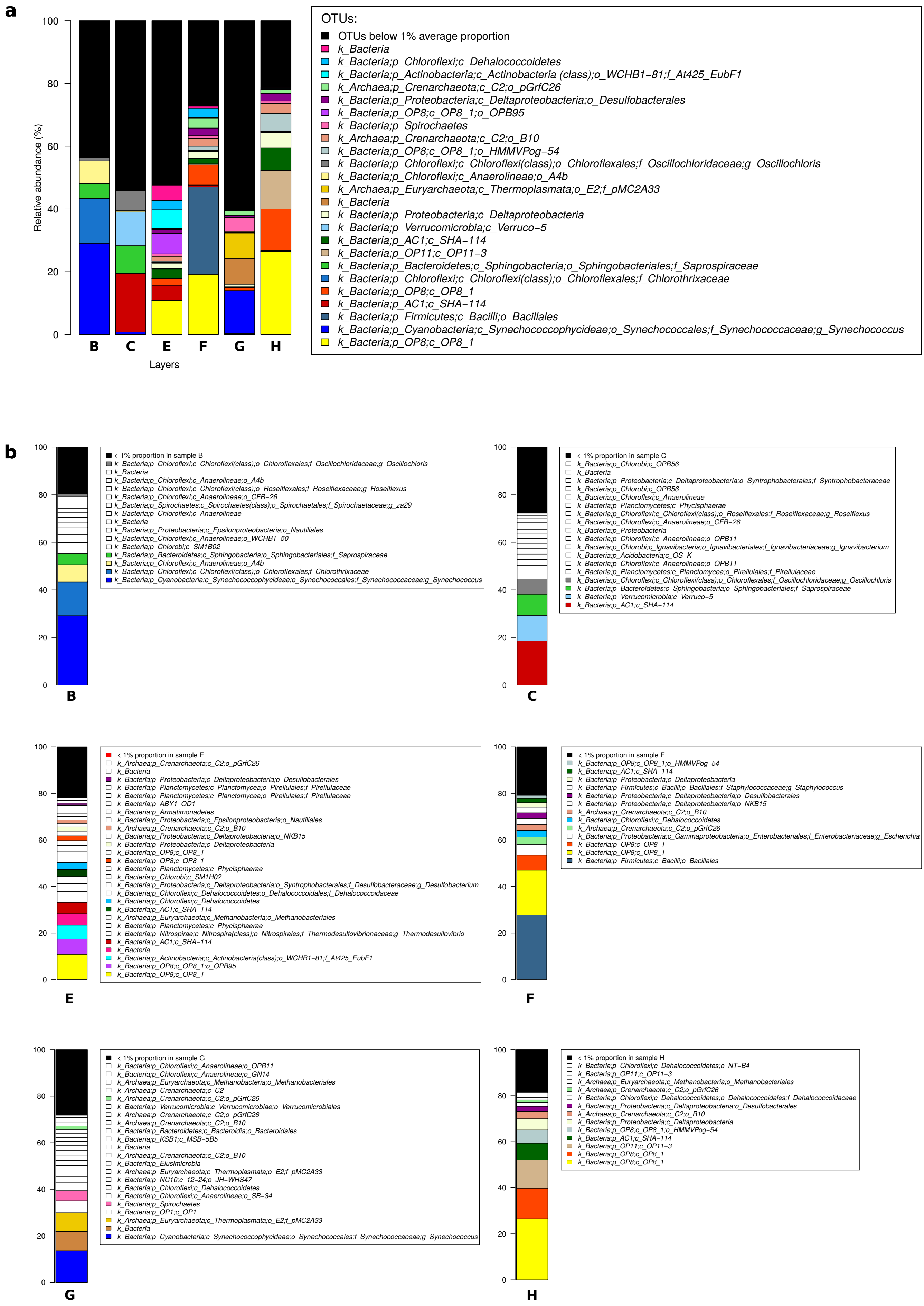

Supplement: Supplementary file 1 — Supplementary Figure S1 [file 41396_2020_705_MOESM1_ESM.pdf]
