# Supplementary material for: Insights into the dynamics between viruses and their hosts in a hot spring microbial mat
Source: ISME J. 2020 Jul 13;14(10):2527–41. doi: 10.1038/s41396-020-0705-4 (PMC7490370; doi:10.1038/s41396-020-0705-4)

Supplementary Figure S2: Number of detected viral-like contigs by the two different pipelines used in this study.

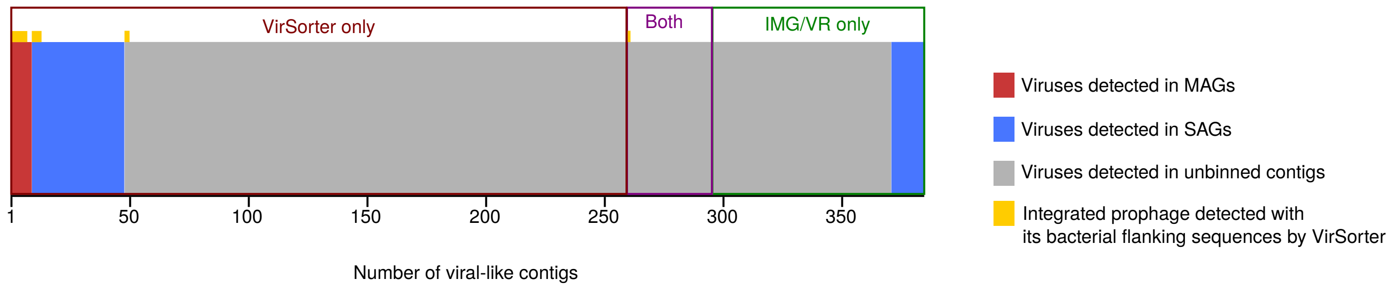

Supplement: Supplementary file 2 — Supplementary Figure S2 [file 41396_2020_705_MOESM2_ESM.pdf]
