# Supplementary material for: Insights into the dynamics between viruses and their hosts in a hot spring microbial mat
Source: ISME J. 2020 Jul 13;14(10):2527–41. doi: 10.1038/s41396-020-0705-4 (PMC7490370; doi:10.1038/s41396-020-0705-4)

Supplementary Figure S3: Viral clusters and singletons in relation to host taxonomy. The alluvial plot shows the complete set of the 385 detected viral contigs in this study and phylum-level taxonomy of their host, if known. Related Figure 2b shows only a subset of this figure (shows only singletons with assigned host taxonomy and only those clusters which contained some contigs with assigned host taxonomy).

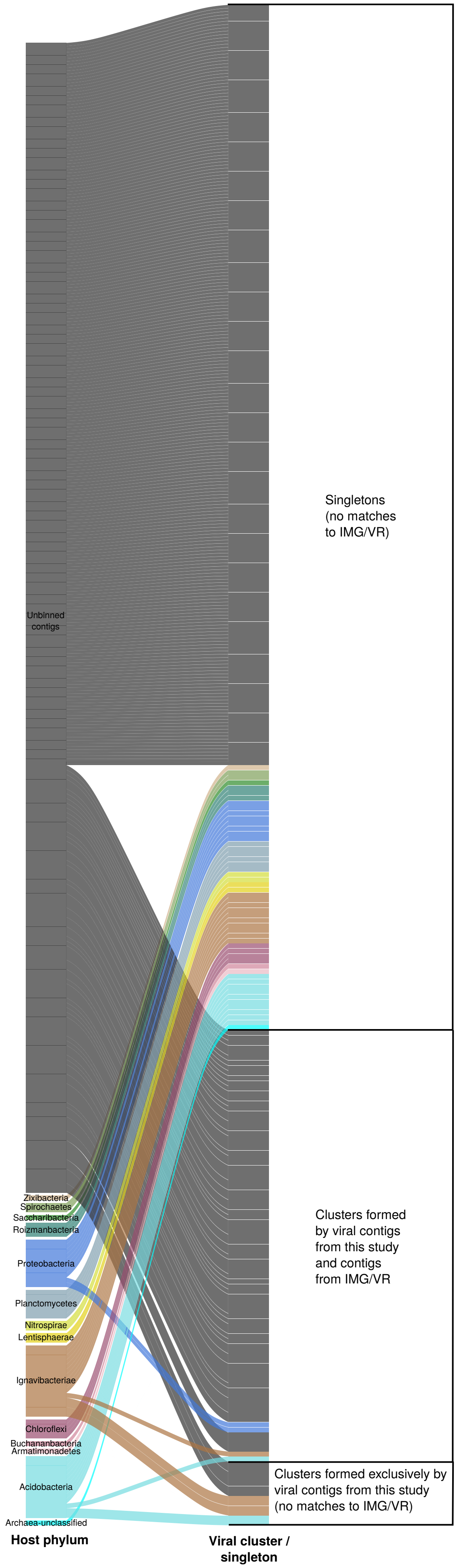

Supplement: Supplementary file 3 — Supplementary Figure S3 [file 41396_2020_705_MOESM3_ESM.pdf]
