# Supplementary material for: Insights into the dynamics between viruses and their hosts in a hot spring microbial mat
Source: ISME J. 2020 Jul 13;14(10):2527–41. doi: 10.1038/s41396-020-0705-4 (PMC7490370; doi:10.1038/s41396-020-0705-4)

Supplementary Figure S4: Detection of presence of actively replicating phages in SAGs by comparing Cp with genome completeness. In SAGs with actively replicatiny phages, the value of crossing point (Cp) of the qPCR used for monitoring the whole genome amplification should be significantly lower than in normal cells, and the genome completeness of the hosts should be also significantly lower. Our data indicate that none of the SAGs with detected viral contigs (red dots) was an outlier compared to the SAGs without viral contigs, indicating that the viruses in the SAGs were integrated prophage. The red line indicates a linear regression fit.

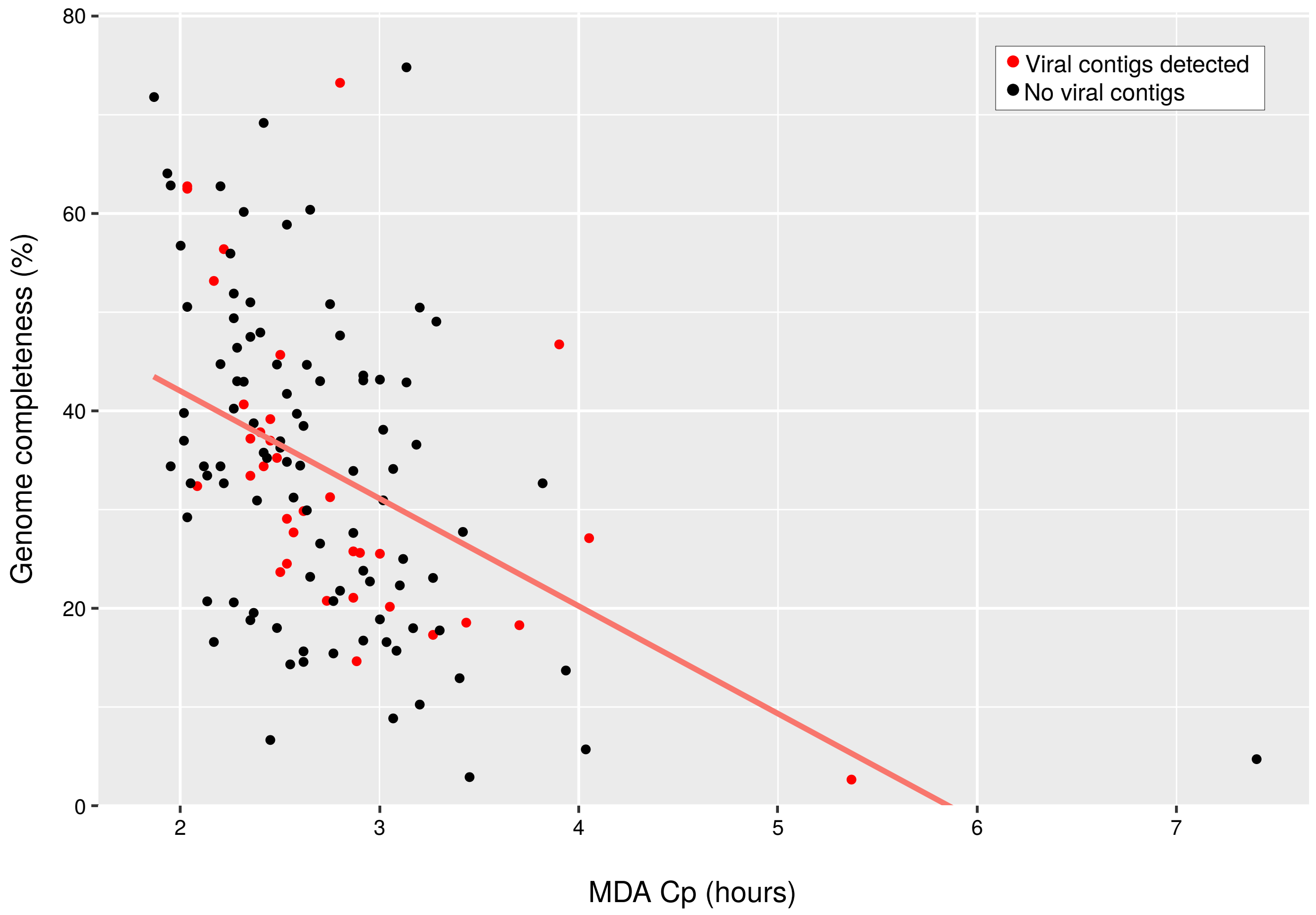

Supplement: Supplementary file 4 — Supplementary Figure S4 [file 41396_2020_705_MOESM4_ESM.pdf]
