# Supplementary material for: Insights into the dynamics between viruses and their hosts in a hot spring microbial mat
Source: ISME J. 2020 Jul 13;14(10):2527–41. doi: 10.1038/s41396-020-0705-4 (PMC7490370; doi:10.1038/s41396-020-0705-4)

Supplementary Figure S5: Detection of presence of actively replicating phages in SAGs by comparing read coverage of contigs. Theoretically, in SAGs with actively replicating phages, viral contig of activately replicating phage can have much higher read coverage than the bacterial contigs within the same SAG, despite MDA bias. Such trend was not observed in our dataset.

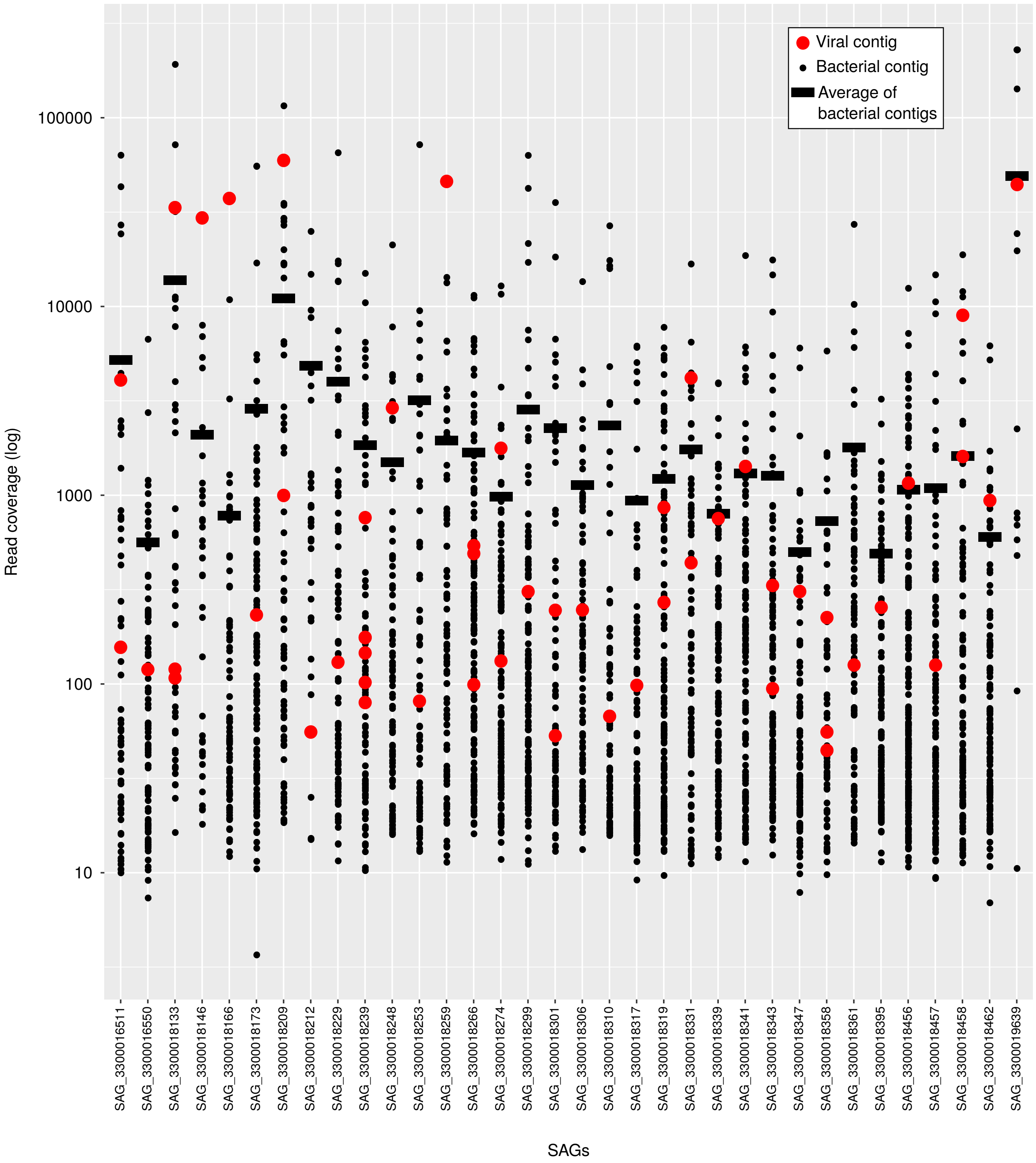

Supplement: Supplementary file 5 — Supplementary Figure S5 [file 41396_2020_705_MOESM5_ESM.pdf]
